# Conservative two-stage revision with primary components of infected total hip arthroplasty: An analysis of survival, clinical and radiographic outcomes

**DOI:** 10.1371/journal.pone.0239981

**Published:** 2020-10-01

**Authors:** Giorgio Burastero, Mattia Alessio-Mazzola, Luca Cavagnaro, Francesco Chiarlone, Giuliana Carrega, Andrea Giorgio Capello, Stefano Lovisolo, Lamberto Felli

**Affiliations:** 1 Bone Infection Unit, Department Orthopaedic and Traumatology 2, Santa Corona Hospital, Pietra Ligure (SV), Italy; 2 Department of Surgical Sciences (DISC), Orthopaedic Clinic, Ospedale Policlinico San Martino, Genova, Italy; 3 Infectious Diseases and Septic Orthopaedic Centre (MIOS), Ospedale Santa Maria di Misericordia, Albenga (SV), Italy; Consorci Parc de Salut MAR de Barcelona, SPAIN

## Abstract

Few studies provide an analysis of conservative two-stage revision of hip periprosthetic joint infection (PJI) and its impact on final outcome. A conservative revision is defined when soft tissues and bone quality enable the use of primary prosthetic components. Data of patients treated for chronic hip PJI who underwent two-stage revision between 2009 and 2016 and had a minimum of 2 years of follow-up were collected. Oxford Hip Score (OHS), Harris Hip Score (HHS) and radiological and microbiological data were retrieved and analysed. Clinical and functional outcome, survival, mortality, eradication, reinfection and re-revision rates within subgroups of patients with primary components and revision components are reported herein. A total of 148 patients underwent two-stage hip exchange with a mean follow-up of 55.6 ± 23.1 months and a mean age at surgery of 64.3 ± 12.7 years. Forty-four percent of patients underwent conservative revision. The mean HHS significantly improved from 40.6 ± 9.4 points to the final value of 87.8 ± 10.5 points (p = .002), and the mean OHS went from 20.3 ± 3.8 points to 40.3 ± 5. points (p< .001). Patients who were treated with primary components or isolated revision stems in the second stage had a significant reduction in surgical times (p< .001). The mortality rate for all causes of death was 6.8%, the eradication rate was 89.9%, the reinfection rate was 4.7% and the reoperation rate was 7.4% without differences between conservative and non-conservative revisions. Two-stage exchange arthroplasty for total hip arthroplasty (THA) PJI is a good strategy that provides satisfactory results, high eradication rates and no further need for revision. The conservative two-stage revision in patients with adequate bone stock represents a feasible option with good results and survival rates.

## Introduction

Periprosthetic joint infection (PJI) is one of the most common and devastating complications after total hip arthroplasty (THA), occurring in approximately in 1% of cases [1, 2]. The two-stage exchange have high success rate ranging from 85 to 100% [3–6]. Nevertheless, the explantation during the first-stage represents a technical challenge especially with regard to femoral and acetabular bone stock. A conservative revision is defined when soft tissues and bone quality enable the use of primary prosthetic components. The main advantages of standard implants in revision surgery are the reduced invasiveness, the shorter operative times and preserved bone stock. Nonetheless, in case of severe bone deficit, a second-stage reconstruction could be extremely difficult to manage with standard implants and revision components are required [7–9].

Several studies have been conducted on the management of PJI [10, 11] but few provide any data on conservative two-stage revisions and detailed aspects of the type of components that were used for the second stage revision of hip PJI.

The aim of the present study is to provide data on the clinical, radiological and microbiological outcomes of patients treated for PJI after total hip replacement with conservative two-stage exchange with primary components. Moreover, we will analyse the complication rates, reoperations, re-revisions, survivorship and features of the components that were used during the second stage and their relative impact on the clinical outcome of patients with a minimum two-year follow-up.

## Materials and methods

Data for this retrospective, single-centre study were collected using the prospective institutional arthroplasty registry.

This study was approved by the Institutional Review Board, and all included patients gave written informed consent to analysis of their medical records and data collection in the setting of this study.

### Studied population

Patients who underwent two-stage exchange (i.e., resection arthroplasty and spacer followed by reimplantation) for primary hip PJI between January, 2009 and January, 2016 were eligible for the study. PJI was diagnosed using MSIS criteria [12].

Exclusion criteria were: patients without a primary infected prosthesis, patients who had already undergone revision surgery or DAIR for infection, patients treated for the first-stage process in another institution, patients who did not undergo the second stage (reimplantation) for any reason including retained spacer, arthrodesis, Girdlestone arthroplasty, amputation or death occurring within the inter-stage period. Patients without at least a 2-year follow-up were excluded as well.

A total of 253 patients were treated for hip PJI by two-stage revision arthroplasty.

The indication for revision total hip arthroplasty (rTHA) was chronic PJI according to the Zimmerli classification [12–14].

A senior surgeon (G.B.) who is experienced in complex revision arthroplasty performed all the surgical procedures reported in the present study. A two-stage revision was completed in all cases.

### Antibiotic and surgical approach

At the time of explantation, surgical debridement and mobile antibiotic-loaded spacer implantation for the femur (Tecres vancogenx preformed spacer with Vancomycin and Gentamicin—Tecres S.P.A.–Via A Doria, 6–37066 Sommacampana (VR) Italy) and acetabulum [15] were performed.

Three to six intra-operative biopsies for microbiological analysis were obtained by default.

Lateral femoral window osteotomy [16] was performed in patients with well-fixed stems and/or cemented implants. Sonication of the infected implant was routinely performed. An intra-articular surgical drain was used until the second postoperative day.

Specific intravenous antibiotics were routinely administered for at least 4 weeks, followed by oral administration lasting at least 2 weeks. When intraoperative cultures were negative, intravenous administration of glycopeptides and fluoroquinolones was protracted for 3 weeks and was followed by oral fluoroquinolones for 4 more weeks. All patients underwent a washout period without any antibiotic therapy of at least 2 weeks before undergoing preoperative second stage screening for infection (Serum CRP and ESR, Synovial White Blood Cell count and percentage, Synovial Leukocyte esterase, synovial fluid culture and Alpha defensing in doubtful cases).

At the time of re-implantation, new surgical debridement and spacer sonication were carried out. Furthermore, three to six culture samples and 1 specimen for definitive histological examination and frozen section were collected.

All the reimplantation procedures were performed through the posterolateral approach. A surgical drain was left in place until the second postoperative day.

The prosthetic components that were used during the second stage are reported in detail and analysed in the setting of the present study.

Conservative revision was defined as a two-stage procedure using primary acetabular cups and stems. Indications for conservative revision were acetabular Paprosky type I or IIA-IIB and femoral Paprosky type I or II-IIIA bone defects. The Trabecular Metal Monoblock Acetabular Cup System (Zimmer Inc, Warsaw, IN) with highly cross-linked polyethylene liner (Longevity; Zimmer) and the CLS Spotorno Stem (Zimmer, GmbH, Winterthur, Switzerland) were used in all patients underwent conservative two stage revision.

Indication for non-conservative revision were severe bone defects (acetabular Paprosky > IIB and femoral Paprosky > IIIA).

Acetabular revision components included jumbo cups, augments, modular dual mobility cups and custom-made cups, while long monoblock cementless stems (Wagner; Zimmer Inc, Warsaw, IN) were used as revision femoral components. No modular or cemented stems were used.

**Fig 1:**
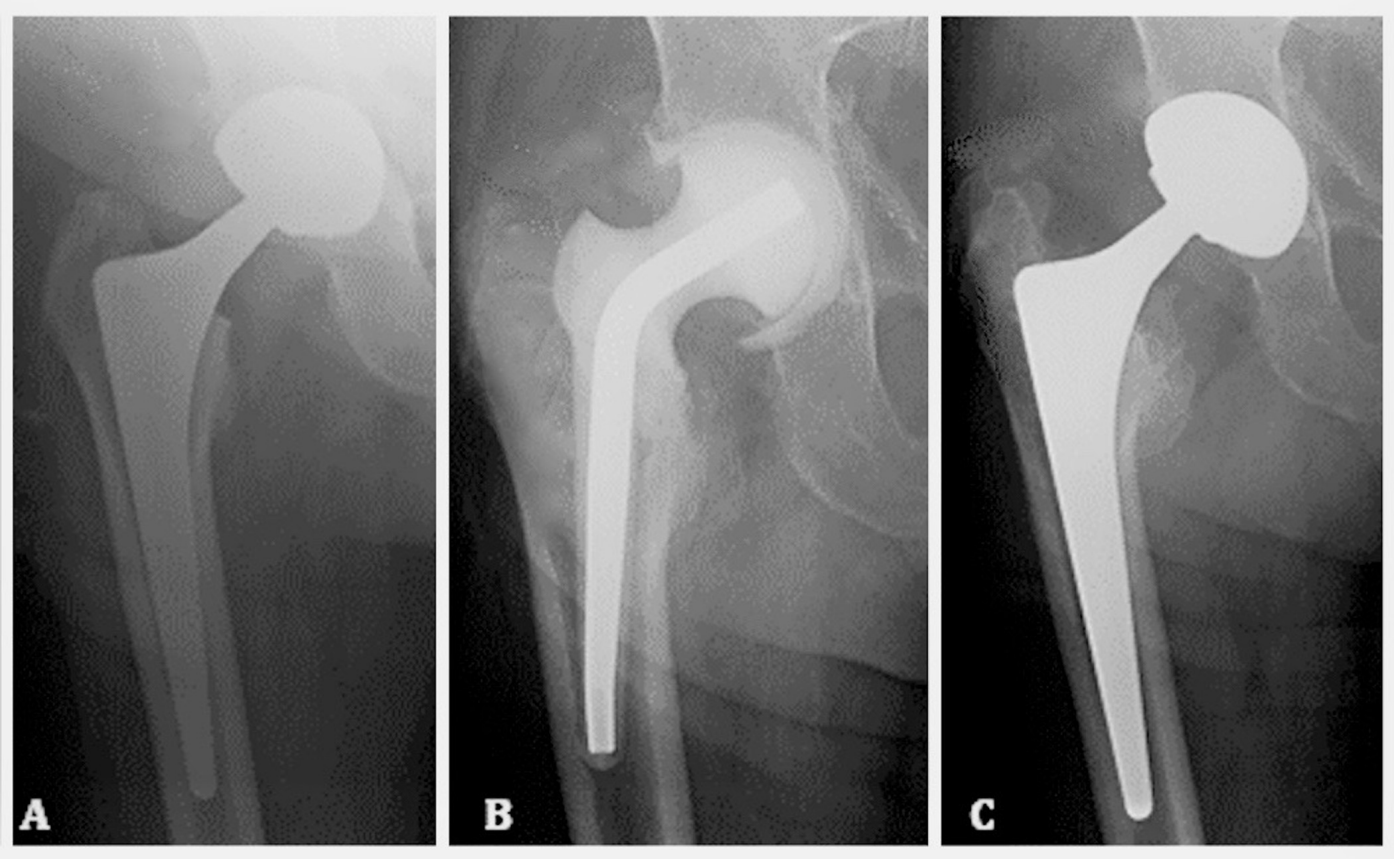
Radiographic example of two-stage revision for hip PJI with primary components. Pre-operative X-ray of infected THA (A), radiographic assessment of spacer placement 6 weeks after the explantation stage (B) and final assessment of completed two-stage procedure at 3 years (C).

Fig 1 shows an example of a two-stage revision that was carried out with primary components.

Patients began ambulation either with partial (50%) or toe-touch weight-bearing and a walker or crutches starting the second postoperative day regardless of the type of prosthetic components that were used.

Supervised physical therapy and continuous passive motion were started the day after surgery and went on for 6 to 8 weeks.

### Follow-up

All patients were assessed clinically and radiographically for a mean of 55.6 ± 23.1 months and none of the patients were lost to follow-up.

Patients without recent follow-up were contacted for the present study.

Patients underwent complications were re-scheduled to be examined with shorter follow up depending on specific condition.

The standard anteroposterior pelvis, frontal hip, and true lateral radiographs were taken one day post-operatively and at 3, 6, and 12 months post-surgery, and annually thereafter.

### Outcomes

The clinical outcome was evaluated by Harris Hip Score (HHS), Oxford Hip Score (OHS) and range of motion at 3, 6, and 12 months after the procedure, and annually thereafter.

The radiographic outcome was assessed evaluating the presence of radiological alterations (loosening, osteolysis, migration, subsidence, cortical hypertrophy or malposition and leg length discrepancy) reviewed by 2 trained orthopaedic fellows (AC, SL). Unclear cases were solved by consensus. Stem alignment was assessed by the axial alignment of the proximal femur on the AP and lateral radiographs.

Malposition was defined when the main axis of the stem differed >5 mm from the anatomic femoral axis. The lateral inclination of the acetabular component was evaluated by defining neutral as an inclination between 30 and 45° [17]. Osteolysis was defined as new, expansive radiotransparent lesions that were not present in the immediate postoperative period. A stem was considered to be “subsided” when the gap between the apex of the great trochanter and the most lateral side of the stem shoulder increased by > 2mm between the immediate postoperative period and the last follow-up evaluation. The pre- and post-operative lateral hip offsets were assessed by digital measurement [18].

Minor complications (wound dehiscence, superficial wound problems) as well as major ones (deep infection, aseptic loosening, intra-operative or post-operative fractures, revision, reoperation) were investigated and fully reported.

Septic recurrence was defined as a secondary infection following the two-stage exchange that was caused by the same pathogens with the same antibiogram. New infection was defined as a secondary infection after the two-stage exchange that was caused by other pathogens or different antibiogram.

Revision was defined as any kind of surgery after the second stage requiring the removal of fixed implant components. Reoperation was defined as any kind of surgical procedure that involved the specific hip joint following the reimplantation, with or without the removal of implant components. Implant survival was defined as the time from two stage to any revision or latest follow-up.

The primary outcome of interest was the difference between conservative and non-conservative revision in mortality, clinical and functional outcome and radiological results in patients underwent staged septic rTHA.

The secondary outcome measures were defined as revision rate for septic recurrence, eradication rate and differences between patients with primary components and patients with revision components.

According to the Diaz-Ledezma criteria [19], successful reimplantation was defined as control of the infection (healed wound without fistula, drainage, or pain), no secondary surgical procedures due to infection after reimplantation surgery (second stage), and no occurrence of PJI-related mortality (i.e., sepsis or necrotizing fasciitis).

### Statistical analysis

Categorical variables were expressed as the absolute number of cases and/or percentage.

The Shapiro-Wilk Test was used to identify normally distributed parameters.

Differences between means were calculated with the T-test for continuous variables or with the Mann-Whitney U test if not normally distributed. The non-parametric Wilcoxon Signed Rank test was used to compare continuous matched pre-operative and final data. Categorical variables were calculated using the Chi-square test or Fisher’s exact test.

The analysis of variance (ANOVA) was used to compare means of continuous normally distributed variables in two or more independent comparison groups. The Kruskal-Wallis test was used for not normally distributed variables.

The non-parametric Spearman’s rho coefficient was used to assess correlation between continuous or ordinal values.

Variables achieving the p value < 0.1 in univariate analysis were examined using multivariate logistic regression analysis and backward selection process. The significance threshold for tests was set at p < .05.

The inter-observer reliability for radiological analysis was evaluated by Cohen’s Kappa coefficient. Kaplan-Meier survival function curves were created using all parameters to analyse survivorship free of revision for any reason for all the implants. A p-value of <0.05 was considered statistically significant.

## Results

A total of 253 patients were screened for study eligibility related to hip chronic PJI between 2009 and 2016. Twenty-three patients did not undergo the two-stage procedure due to comorbidities, complications or death, 34 had undergone the first stage procedure at another institution, 40 had had a previously failed two-stage exchange and 8 were lost to follow-up. A total of 148 patients completed the two-stage hip exchange and their data was retrieved for study evaluation. The mean inter-stage period was 12.7 ± 4.8 weeks. The mean follow-up was 55.6 ± 23.1 (range: 24 to 117; 95% CI: 57.8 to 65.8) months and the mean age of the patients at the time of surgery was 64.3 ± 12.7 (range: 33 to 84; 95% CI: 62.2 to 66.3) years. The mean body mass index (BMI) was 26.5 ± 5.2 (range: 19 to 42.2; 95% CI: 25.3 to 27.7) kg/m2, and 14 (9.5%) patients were classified as obese (BMI> 30).

Eighty patients (54.1%) were female and 68 (45.9%) were male.

Table 1 shows the results of isolated bacteria.

**Table 1:** Microbiological data.

Microbiological cultures	Conservative revision	Non-conservative revision	Total
**Positive cultures**	55	58	113 (76.4)
*Staphylococcus Aureus*	12	24	36
Polymicrobic flora	8	12	20
*Coagulase-negative Staphylococcus*	9	10	19
*Staphylococcus epidermidis*	9	6	15
*Streptococcus spp*.	7	4	11
*Gram Negative*	3	5	8
*Candida spp*.	1	1	2
Others ^a^	1	1	2
**Negative Cultures**	11	24	35 (23.6)
**Total**	66	82	148

^a^ Others bacteria were: *mycobacterium tuberculosis* (1), *Acynetobacter baumanii* (1).

With regard to co-morbidities, 21 patients were diabetic (14.2%), 13 (8.8%) were affected by heart failure and 4 (2.7%) had rheumatoid arthritis. Ninety-seven (65.6%) were non-smokers, 24 (16.2%) were smokers and 27 (18.2%) were former smokers [20].

Sixty-six (44.6%) patients underwent a conservative revision and 82 (55.4%) a non-conservative revision. Details are listed within the Table 2.

**Table 2:** 

	Conservative Revision	95% CI	Non-conservative Revision	95% CI	P value
**Number of patients**	66 (44.6)	-	82 (55.4)	-	-
**Mean follow-up (months)**	53.0 ± 27.2	45.6 to 60.3	52.0 ± 28.5	42.9 to 61.1	.952
**Age at surgery (years)**	63.2 ± 14.1	59.3 to 67.0	61.4 ± 14.4	53.7 to 66.0	.406
**Body Mass Index (mean value)**	27.5 ± 4.8	25.6 to 30.2	24.9 ± 3.6	23.2 to 26.7	.379
**Gender (male/female)**	29/37	-	39/43	-	.741
**Relevant comorbidities**	15 (22.7)	-	23 (28.0)	-	.571
**Previous surgeries**	1.3 ± 0.6	1.2 to 1.4	1.8 ± 0.8	1.5 to 2.0	**.003**
**Operative time (min)**	121.0 ± 50.0	107.6 to 134.4	156.8 ± 45.2	141.9 to 171.6	**< .001**
**Paprosky type I (femur)**	11 (16.7)	-	0	-	**< .001**
**Paprosky type II (femur)**	37 (56.1)	-	0	-	**< .001**
**Paprosky type IIIA (femur)**	18 (27.3)	-	0	-	**< .001**
**Paprosky type IIIB (femur)**	0	-	54 (65.9)	-	**< .001**
**Paprosky type IV (femur)**	0	-	2 (3.6)	-	**.503**
**Paprosky type I (acetabulum)**	5 (7.6)	-	0	-	**.016**
**Paprosky type II (acetabulum)**	61 (91.4)	-	0	-	**< .001**
**Paprosky type III (acetabulum)**	0	-	47 (57.3)	-	**< .001**
**Paprosky type IV (acetabulum)**	0	-	2 (2.4)	-	.503
**Pre-op HHS**	40.6 ± 9.4	39.1 to 42.1	40.6 ± 8.1	38.0 to 43.3	.912
**Final HHS**	88.4 ± 9.2	85.8 to 90.9	87.8 ± 10.5	86.1 to 84.5	.667
**Pre-op OHS**	20.3 ± 3.8	19.7 to 21.0	20.2 ± 3.0	19.3 to 21.2	.810
**Final OHS**	40.4 ± 4.5	39.1 to 41.6	40.3 ± 5.2	38.5 to 41.2	.548
**Pre-operative offset (mm)**	50.9 ± 4.6	49.6 to 52.3	49.4 ± 6.1	47.2 to 51.7	.142
**Final offset (mm)**	52.9 ± 5.7	51.5 to 54.3	49.8 ± 5.1	47.9 to 51.7	**.018**
**Mean offset gain (mm)**	2.0 ± 5.0	0.5 to 3.5	0.4 ± 6.1	-1,9 to 2.6	.441

Table 2 shows the overall features of patients who underwent conservative and non-conservative two-stage rTHA.

During the first stage surgery and the inter-stage period, a 13.5% rate of complications was reported: 7 (4.7%) intra-operative femoral fractures, 7 (4.7%) post-operative spacer dislocations, 3 (2.0%) post-operative femoral fractures, 2 (1.4%) pulmonary embolisms, and 1 (0.7%) infection persistence.

Five (3.4%) reoperations for spacer exchanges were performed during the inter-stage phase due to 3 recurrent dislocations, 1 displaced femur fracture and 1 infection persistence.

### Clinical and functional outcome

A significant improvement in all the evaluated clinical scores was found between pre-operative and final values in both groups.

The mean HHS significantly improved from 40.6 ± 9.4 (range: 25 to 83; 95% CI: 39.1 to 42.1) points to the final value of 87.8 ± 10.5 (range: 43.5 to 100; 95% CI: 86.1 to 89.5) points (p = .002), and the mean OHS went from 20.3 ± 3.8 (range: 12 to 34; 95% CI: 19.7 to 21.0) points to 40.3 ± 5.2 (range: 17 to 48; 95%CI: 39.5 to 41.2) points (p< .001).

No significant differences between pre-operative HHS (p = .229), pre-operative OHS (p = .265) and final HHS (p = .097) scores within conservative and non-conservative groups were observed. However, patients that underwent conservative revisions had had significantly fewer previous surgeries (p< .003) and lower Paprosky bone loss grade at second stage (Table 2).

**Fig 2:**
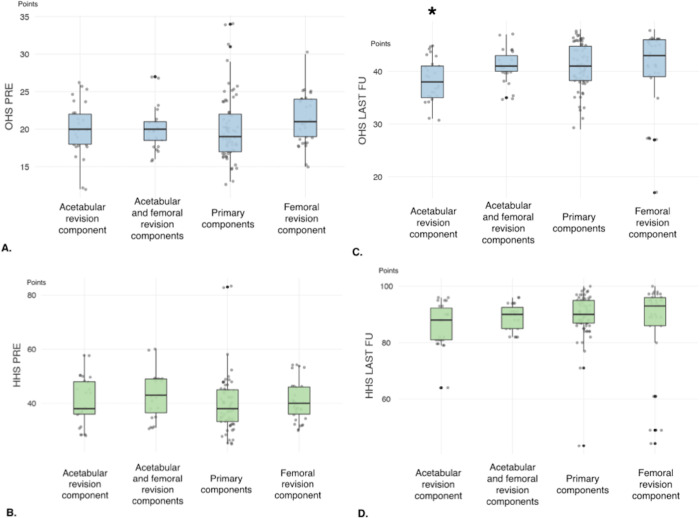
Graph presentation of clinical scores. Pre-operative OHS (A), HHS (B) and final values of OHS (C) and HHS (D) within groups of patients sub-divided by type of component used in the second stage procedure. Asterisks highlight the significant data that were identified by the analysis of variance (ANOVA) and post hoc test.

Patients with isolated acetabular revision components had lower final OHS scores than patients with primary components (p = .009), revision stems (p = .016) and acetabular and femoral revision components (p = .036). (Fig 2).

**Fig 3:**
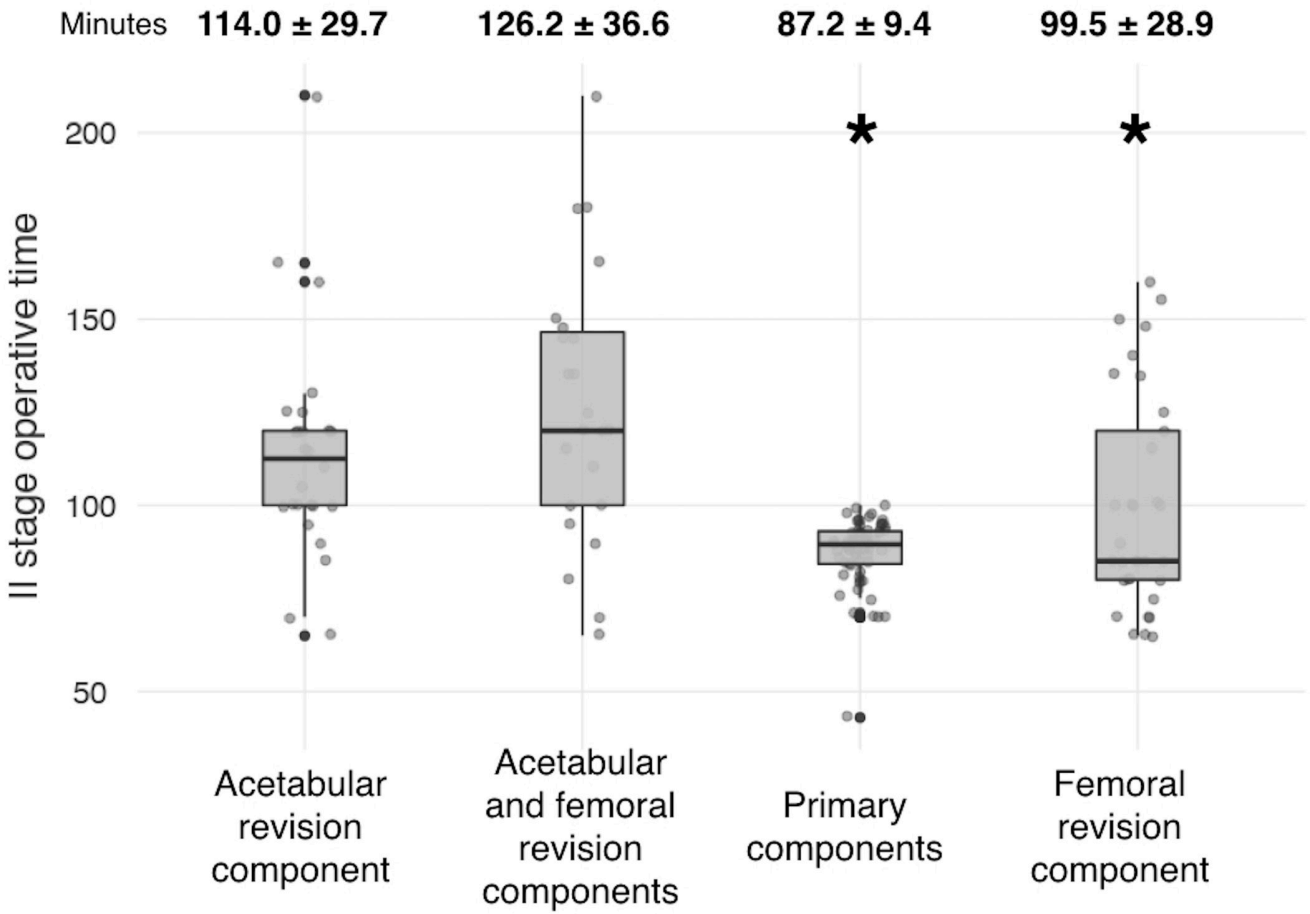
Graph and numerical presentation of mean surgical times with standard deviations within groups of patients sub-divided by type of component used in second stage procedures. Asterisks highlight the significant data identified by ANOVA and post hoc test.

The type of prosthetic components that were used during the second stage procedures significantly influenced the operative time (p< .001). Both subgroups of patients who underwent conservative second stage revision or second stage surgery with revision stems and primary cups had significantly shorter surgical times (p< .05) compared to other subgroups. Details concerning the duration of second stage surgeries are shown in Fig 3.

The type of prosthetic components (revision or primary) that were used for second stage surgeries did not influence mortality, eradication, reinfection or re-operation rates (p> .05).

### Radiographic evaluation

At the patients' most recent follow-up evaluations, X-rays demonstrated a 97.3% rate of good anteroposterior and lateral positioning, while 4 outliers were observed, two of which were in varus stem positioning (both primary stems) and two in horizontal cup positioning (dual mobility cups). No differences were found between conservative and non-conservative rTHA. One patient of conservative group had femoral diaphyseal cortical hypertrophy and occasional tight pain with mild functional limitation.

Eleven patients showed radiolucent lines of less than 1 mm after the implantation with no progression over time. Six of them were on the femoral side (5 in Gruen’s zone 6 on the anteroposterior view and in zone 13 on the lateral view, and 1 in zones 2–6 on the anteroposterior view and zones 9–13 on the lateral view) and seven of them were on the acetabular side (6 in DeLee-Charnley zones 1–2, and 1 in zone 1).

Heterotopic ossifications were observed in 17 patients (12 Brooker grade II and 5 Brooker grade III).

No statistically significant associations were found when we compared radiolucency, ossification, leg length discrepancy and malalignment between primary and revision components.

The inter-observer reliability for parameters of radiographic assessment (osseointegration, migration, loosening, osteolysis, cortical hypertrophy, malposition) was 0.87, 0.93, 0.95, 0.88, 0.97 and 0.83, respectively, showing almost unanimous agreement between the 2 observers.

### Mortality

**Fig 4:**
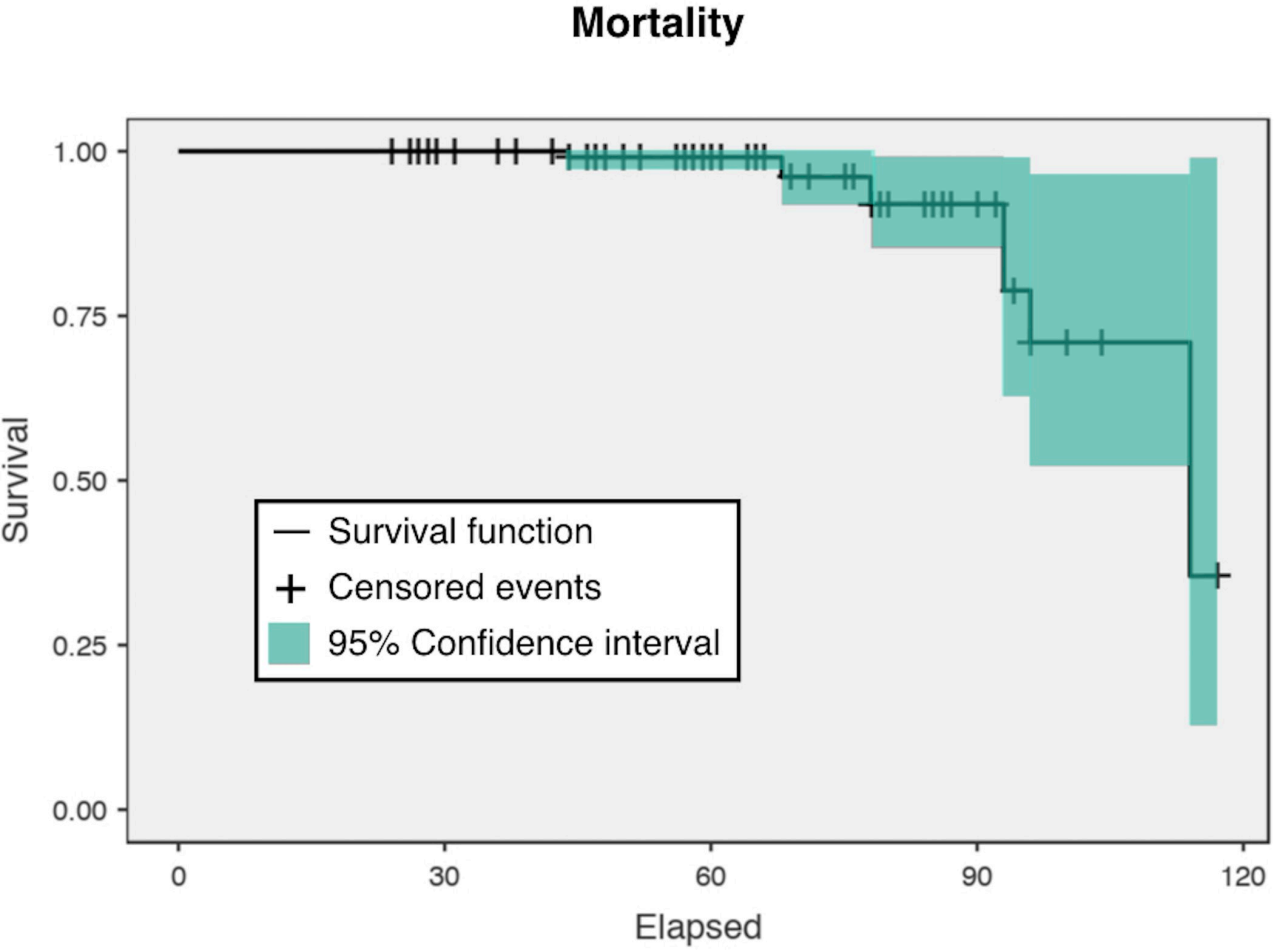
Kaplan-Meier survival function for mortality in patients (n = 148) treated with two-stage revision. The 95% confidence interval is shown in green.

The mortality rate in the present study for all causes of death was 6.8% (with a 1.4% prevalence of sepsis) with no differences between conservative and non-conservative rTHAs (p> .05) (Table 3). Fig 4 shows the overall survival rate of the entire study population.

**Table 3:** Results of two-stage revision for hip PJI.

	Conservative	Non-conservative	P value	Total
**Mortality**	6 (9.1)	4 (4.9)	.342	10 (6.8)
Tumor	3 (4.5)	2 (2.4)	.656	5 (3.4)
Heart failure	2 (3.0)	1 (1.2)	.586	3 (45.9)
Sepsis	1 (1.5)	1 (1.2)	1.000	2 (1.4)
**Eradications**	56 (84.8)	77 (93.9)	.099	133 (89.9)
**Reinfection**	5 (7.6)	2 (2.4)	.243	7 (4.7)
**Intraoperative complications**				
Femoral fracture	1 (1.5)	1 (1.2)	1.000	2 (1.4)
**Postoperative complications**	8 (12.1)	6 (7.3)	.401	14 (9.5)
Reinfection	5 (4.5)	2 (2.4)	.243	7 (4.7)
Dislocation	3 (4.5)	0 (0)	.051	3 (2.0)
Loosening	0 (0)	3 (3.7)	.254	3 (2.0)
Periprosthetic fracture	0 (0)	1 (1.2)	1.000	1 (0.7)
**Reoperations**	6 (9.1)	5 (6.1)	.544	11 (7.4)
Component exchange	3 (4.5)	2 (2.4)	.656	5 (3.4)
Two-stage re-revision	2 (3.0)	1 (1.2)	.586	3 (2.0)
Girdlestone	1 (1.5)	1 (1.2)	1.000	2 (1.4)
Osteosynthesis	0 (0)	1 (1.2)	1.000	1 (0.7)

The significant factors influencing mortality according to univariate analysis were Gram negative infections (odds ratio 3.1; 95% CI: 0.7 to 13.5; p< .001), age at surgery (≥ 70 years; odds ratio: 18.6; 95% CI: 2.3 to 151; p< .001; ≥ 80 years; odds ratio: 239; 95% CI: 25.2 to 2273) and non-eradication of infection (odds ratio: 7.7; 95% CI: 1.9 to 31.4; p = .001).

None of the following were found to be significant factors; positive cultures at second stage (p = .779), polymicrobial infections (p = .114), relevant comorbidities (p = .125), type of prosthetic implant (p = .086).

The overall model test was found to be significant by multivariate analysis (p< .001). Age at surgery (p = .001) and non-eradication of infection (p = .020) were both significantly independent factors correlated to mortality.

### Eradication of infection

The eradication rate in the present series was 89.9% with 15 patients receiving suppressive therapy, with no significant differences between conservative and non-conservative rTHAs (p> .05) (Table 3). Thirteen patients had clinically controlled infection after three months of antibiotic therapy and 2 patients required a second two-stage procedure due to septic recurrence.

When microbiological findings were analysed by univariate analysis, we found that Gram negative (odds ratio: 11.7; 95% CI: 2.6 to 53.4; p< .001) and polymicrobial infections (odds ratio: 5.7; 95% CI: 1.8 to 18.3; p = .002) had a significant influence on the eradication rate.

Multivariate analysis confirmed that Gram negative (p< .001) and polymicrobial infections (p< .001) were both significantly associated with non-eradication of the infection.

### Reinfections

The reinfection rate in this study was 4.7% without any significant differences between conservative and non-conservative revisions (p> .05) (Table 3). The isolated bacteria were: Gram negative (n = 4), Gram positive (n = 1) and polymicrobial infections (n = 2).

The Gram negative infections included *Pseudomonas aeruginosa* (n = 1) and *Escherichia coli* (n = 3), while the isolated Gram positive infection was *MRSA* (n = 1).

Of the 7 patients with reinfection, 3 died during follow-up (2 of sepsis and 1 of a metastatic pancreatic tumour).

Five patients underwent a re-revision for reinfection (3 underwent a second two-stage procedure and 2 had Girdlestone arthroplasty for relevant comorbidities thus precluding a second two-stage procedure).

Details of outcome are reported in Table 3.

We found that age at surgery >80 years (odds ratio: 5.8; 95% CI: 1.3 to 26.5; p = .012), Gram negative infections (odds ratio: 27.0; 95% CI: 5.2 to 140; p< .001) and polymicrobial infections (odds ratio: 6.2 95% CI: 1.5 to 25.3; p< .001) significantly influenced the reinfection rate.

On the contrary, the type of prosthetic components (p = .321), the presence of comorbidities (p = .054), and the operative time of the second stage procedure (p = .133) did not significantly influence the reinfection rate.

After multivariate analysis was applied, the regression model showed that infections sustained by Gram negative (p< .001) and polymicrobial flora (p = .003) were both significantly correlated with reinfection.

### Reoperations/revisions

The overall reoperation rate was 7.4%, with a 6.8% rate of revision with no differences between conservative and non-conservative rTHAs (p> .05) (Table 3).

The causes of reoperation were: new infection (n = 3), loosening (n = 3), septic failure (n = 2), recurrent dislocation (n = 2) and periprosthetic Universal Classification System type C fracture (n = 1).

**Fig 5:**
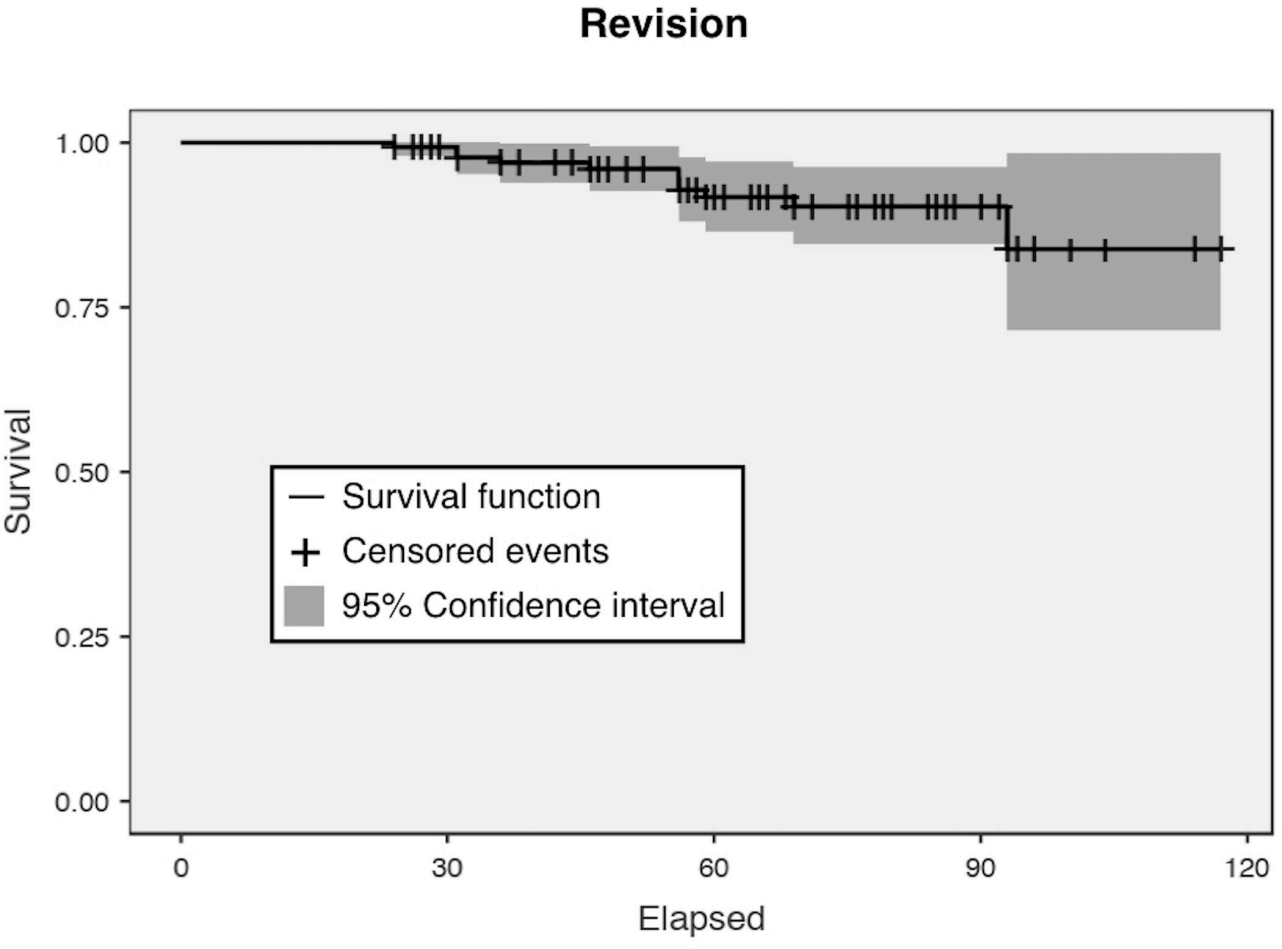
Kaplan-Meier survival function for the survival of the population (n = 148) free of revision for any cause. The 95% confidence interval is shown in grey.

Fig 5 shows the reoperation survival function of the entire population.

We found that dislocation (odds ratio: 15; 95% CI: 1.9 to 119; p = .028), loosening (odds ratio: 113; 95% CI: 5.4 to 2375; p< .001), polymicrobial infection (odds ratio: 16.7; 95% CI: 4.3 to 64.7; p< .001) and Gram negative infection (odds ratio: 9.9; 95% CI: 2 to 49; p< .001) were risk factors for reoperation after two-stage rTHA.

Age at surgery (p = .090) and type of implant used in the second stage (p = .104) were not significant risk factors correlated to reoperation.

Multivariate analysis highlighted that polymicrobial infections (p< .001) and Gram negative infections (p< .001) were significantly associated with reoperation, while regression analysis did not confirm that dislocation and loosening were significantly associated.

## Discussion

The main finding of the present study is that conservative two-stage revision of infected THR shows good clinical and radiological outcome and high rates of eradication and implant survival at midterm follow-up.

Due to the progressively increasing trend of revisions in ever-younger patients [21], nowadays greater attention is being paid to component selection and “de-escalation” surgery [22]. Casella et al. [23] presented their results regarding twenty-one consecutive revision procedures involving conservative hip arthroplasty using cementless primary components. Nevertheless, the series included a limited number of septic cases and it focused on the revision of conservative primary arthroplasties.

To our knowledge, no previous studies have reported the clinical and radiological outcomes of large series of conservative two-stage rTHAs.

In their systematic review of the literature, Cavagnaro et al. [24, 25] highlighted that femoral cementless revision is a feasible option in Paprosky type I and II defects, and reported a survival rate of 95.6% after 4.7 years of follow-up. However, few data on conservative primary acetabular component selection are available in the current literature.

The present study showed that 44.6% of patients who completed the two-stage exchange for hip PJI were treated with conservative revision and primary components. The advantage of a primary implant is the decreased operative time without any significant increase in the failure rates. In spite of these findings, the present study highlighted more dislocations in patients treated with conservative revisions (4.5% vs 0%) without any significant increase in reoperations and revisions. The clinical outcomes of patients with conservative and non-conservative revision were also somewhat comparable, with the exception of OHS results. Patients with isolated acetabular revision components had lower OHS scores at final follow-up. It is not surprising that the poor subjective clinical outcome might be due to the severity of the bone loss at the acetabular side, to the multiple previous surgeries and to the altered hip biomechanics of these patients [26, 27]. Data confirmed that patients with revision components had significantly more previous surgeries than patients with primary components (p = .003).

Several outcome measures and risk factors have been analysed in the present study.

Gram negative and polymicrobial infections have been identified as factors of worse outcome. These pathogens are especially challenging in septic revisions due to the small number of effective antibiotics, the high frequency of Multidrug Resistance (MDR), Gram-negative bacilli especially in southern European regions and low drug bioavailability within the bony tissue [28, 29].

The presence of comorbidities has been identified as a significant risk factor for worse outcome and increased mortality in two-stage revisions by several authors [28, 29]. However, univariate and multivariate analysis of the present study did not confirm this aspect, although the data nearly reached significance. This can probably be explained by the high prevalence of comorbidities in our population (64.2%).

The mortality rate in the present study was 6.8% which is comparable to rates that have been reported in other studies ranging from 2.9 to 19% [30, 31], thus confirming that the two-stage procedure for infected THR has a remarkable mortality rate. Moreover, we have to consider that not all patients with infected THRs concluded the two-stage procedure due to related comorbidities.

This study has several limitations: first, it is a retrospective study with relatively short follow-up. Second, the exclusion of patients who did not complete the two-stage procedure represents a selection bias that influences the final results. Third, the lack of anaesthesiologic data precluded the stratification of patients, and the scoring or indexing of related comorbidities could have skewed the results and the relative impact of comorbidities on the final outcome.

## Conclusion

Conservative two-stage exchange arthroplasty for THA PJI represents a good strategy with satisfactory results and high eradication rate and survival. The use of primary components in two-stage revision in patients with adequate bone stock represents a feasible option providing good results and survival rates.

## Supporting information

S1 Data (XLSX)

## References

[1] Pulido L, Ghanem E, Joshi A, Purtill JJ, Parvizi J. Periprosthetic joint infection: the incidence, timing, and predisposing factors. Clin Orthop 2008;466: 1710e5. 10.1007/s11999-008-0209-4 PMC2505241 18421542

[2] Carrega G, Bartolacci V, Burastero G, Casalino Finocchio G, Grappiolo G, Salomone C, et al Etiology of prosthetic joint infections in a tertiary care centre in Italy. Infez Med. 2008 12;16(4):204–8. 19155685

[3] Cooper HJ, Della Valle CJ. The two-stage standard in revision total hip replacement. Bone Joint J. 2013 11;95-B (11 Suppl A):84–7. 10.1302/0301-620X.95B11.32906 24187360

[4] Lim SJ, Park JC, Moon YW, Park YS. Treatment of periprosthetic hip infection caused by resistant Microorganisms using 2-stage reimplantation protocol. J Arthroplasty 2009;24:1264e9. 10.1016/j.arth.2009.05.012. 19523784

[5] Volin SJ, Hinrichs SH, Garvin KL. Two-stage reimplantation of total joint in- fections. Clin Orthop Relat Res 2004;427:94e100. 10.1097/01.blo.0000143559.34143.3d. 15552143

[6] Chen AF, Heller S, Parvizi J. Prosthetic joint infections. Surg Clin North Am 2014; 94:1265e81. 10.1016/j.suc.2014.08.009. 25440123

[7] Hsieh PH, Shih CH, Chang YH, Lee MS, Yang WE, Shih HN. Treatment of deep infection of the hip associated with massive bone loss: two-stage revision with an antibiotic-loaded interim cement prosthesis followed by reconstruction with allograft. J Bone Joint Surg Br. 2005; 87:770–775. 10.1302/0301-620X.87B6.15411 15911656

[8] Lombardi AV Jr, Berend KR. The shattered femur: radical solution options. J Arthroplasty. 2006;21(Suppl 1):107–111 16781442 10.1016/j.arth.2006.01.007

[9] Burastero G, Cavagnaro L, Chiarlone F, Alessio-Mazzola M, Carrega G, Felli L. The Use of Tantalum Metaphyseal Cones for the Management of Severe Bone Defects in Septic Knee Revision. J Arthroplasty. 2018 12;33(12):3739–3745. 10.1016/j.arth.2018.08.026 30266325

[10] Dwyer MK, Damsgaard C, Wadibia J, Wong G, Lazar D, Smith E et al Laboratory Tests for Diagnosis of Chronic Periprosthetic Joint Infection Can Help Predict Outcomes of Two-Stage Exchange.J Bone Joint Surg Am. 2018 6 20;100(12):1009–1015. 10.2106/JBJS.17.00599 29916927

[11] Hofmann AA, Goldberg TD, Tanner AM, Cook TM. Ten-year experience using an articulating antibiotic cement hip spacer for the treatment of chronically infected total hip. J Arthroplasty. 2005 10;20(7):874–9. 10.1016/j.arth.2004.12.055 16230238

[12] Parvizi J, Gehrke T. Definition of periprosthetic joint infection. International Consensus Group on Periprosthetic Joint Infection. J Arthroplasty. 2014 7;29(7):1331 10.1016/j.arth.2014.03.009 24768547

[13] Zimmerli W, Trampuz A, Ochsner PE. Prosthetic-Joint Infections. The diagnosis of PJI was made by a team of experienced infectivologists following the modified diagnostic criteria of Musculoskeletal Infection Society (MSIS) N Engl J Med 2004; 351:1645–1654. 10.1056/NEJMra040181 15483283

[14] Workgroup Convened by Musculoskeletal Infection Society. New definition for periprosthetic joint infection. J Arthroplasty 2011 12;26(8):1136 10.1016/j.arth.2011.09.026 22075161

[15] Burastero G, Basso M, Carrega G, Cavagnaro L, Chiarlone F, Salomone C, et al Acetabular spacers in 2-stage hip revision: is it worth it? A single-centre retrospective study. Hip Int. 2017 3 31;27(2):187–192. 10.5301/hipint.5000446 27886355

[16] Cameron HU. Femoral windows for easy cement removal in hip revision surgery. Orthop Rev. 1990;19:909–912. 2250996

[17] Callanan M C, Jarrett B, Bragdon C R, Zurakowski D, Rubash H E, Freiberg AA, et al The John Charnley Award: risk factors for cup malpositioning: quality improvement through a joint registry at a tertiary hospital. Clin Orthop Relat Res 2011; 469 (2): 319–29. 10.1007/s11999-010-1487-1 20717858 PMC3018230

[18] Dastane M, Dorr LD, Tarwala T, Wan Z, Hip Offset in Total Hip Arthroplasty: Quantitative Measurement with Navigation. Clin Orthop Relat Res. 2011 2; 469(2): 429–436. 10.1007/s11999-010-1554-7 20844997 PMC3018189

[19] Diaz-Ledezma C, Higuera CA, Parvizi J. Success after treatment of periprosthetic joint infection: a Delphi-based international multidisciplinary consensus. Clin Orthop Relat Res 2013;471:2374e82. s11999-013-2866-1. 10.1007/s11999-013-2866-1 PMC3676607 23440616

[20] Gellman MD, Turner JR Encyclopedia of Behavioral Medicine ISBN: 978-1-4419-1004-2 (Print) 978-1-4419-1005-9 (Online) pp 741–742.

[21] Iorio R, Robb WJ, Healy WL, Berry DJ, Hozack WJ, Kyle RF, et al Orthopaedic surgeon workforce and volume assessment for total hip and knee replacement in the United States: preparing for an epidemic. J Bone Joint Surg Am 2008;90: 1598e605. 10.2106/JBJS.H.00067 18594111

[22] Vivès P, Plaquet JL, Leclair A, Blejwas D, Filloux JF. Revision of interlocking rod for loosening of THP. Concept preliminary results. Acta Orthop Belg 1992 58:28–35. 1561868

[23] Casella F, Favetti F, Panegrossi G, Papalia M, Falez F. A new classification for proximal femur bone defects in conservative hip arthroplasty revisions. Int Orthop. 2018 12 11 10.1007/s00264-018-4229-8 30539217

[24] Cavagnaro L, Formica M, Basso M, Zanirato A, Divano S, Felli L. Femoral revision with primary cementless stems: a systematic review of the literature. Musculoskelet Surg. 2018 4;102(1):1–9. 10.1007/s12306-017-0487-7 28669102

[25] Cavagnaro L, Chiarlone F, Divano S, Capello AG, Felli L, Burastero G. Primary cementless stems in septic hip revision: Indications and results. J Orthop Surg (Hong Kong). 2019 May-Aug;27(2):2309499019853999. 10.1177/2309499019853999 31177970

[26] Lachiewicz PF, Soileau ES. Fixation, survival, and dislocation of jumbo acetabular components in revision hip arthroplasty. J Bone Joint Surg Am. 2013 3 20;95(6):543–8. 10.2106/JBJS.L.00758 23515989

[27] McLaughlin JR, Lee KR. Acetabular Revision Arthroplasty Using an Uncemented Deep Profile Jumbo Component: A Ten to Sixteen Year Follow-Up Study. J Arthroplasty 2018 33(2): 496–499. 10.1016/j.arth.2017.09.002 28993083

[28] Ascione T, Pagliano P, Balato G, Mariconda M, Rotondo R, Silvano Esposito S Oral Therapy, Microbiological Findings, and Comorbidity Influence the Outcome of Prosthetic Joint Infections Undergoing 2-Stage Exchange. J Arthroplasty 32 (2017) 2239e2243. 10.1016/j.arth.2017.02.057 28372916

[29] Fagotti L, Tatka J, Costa Salles MJ, Queiroz MC Risk Factors and Treatment Options for Failure of a Two-Stage Exchange Curr Rev Musculoskelet Med. 2018 9; 11(3): 420–427. 10.1007/s12178-018-9504-1 29934884 PMC6105486

[30] Sanchez-Sotelo J, Berry DJ, Hanssen AD, Cabanela ME. Midterm to long-term followup of staged reimplantation for infected hip arthroplasty. Clin Orthop Relat Res 2009;467:219–224. 10.1007/s11999-008-0480-4 18813895 PMC2600996

[31] Hofmann AA, Goldberg TD, Tanner AM, Cook TM. Ten-year experience using an articulating antibiotic cement hip spacer for the treatment of chronically infected total hip. J Arthroplasty 2005;20:874–879. 10.1016/j.arth.2004.12.055 16230238

